# Extender development for optimal cryopreservation of buck sperm to increase reproductive efficiency of goats

**DOI:** 10.3389/fvets.2025.1554771

**Published:** 2025-04-02

**Authors:** Mustafa Bodu, Mustafa Hitit, Olivia Chika Greenwood, Raheem Davian Murray, Erdogan Memili

**Affiliations:** ^1^Cooperative Agricultural Research Center, College of Agriculture, Food, and Natural Resources, Prairie View A&M University, Prairie View, TX, United States; ^2^Department of Reproduction and Artificial Insemination, Faculty of Veterinary Medicine, Selcuk University, Konya, Türkiye

**Keywords:** sperm, cryopreservation, extender, antioxidants, omics, sustainable goat production

## Abstract

Preservation of sperm significantly contributes to the advancement of assisted reproductive technologies, genetic conservation and improvement efforts, and precision breeding of livestock. This review distills knowledge from the existing information and emerging patterns in the field of buck sperm cryopreservation. The primary focus is on the challenges and opportunities associated with improving extender formulations and freezing techniques in order to enhance the vitality of sperm after thawing and to increase the potential for conception. This review assesses the efficacy and limitations of conventional extenders derived from egg yolk or soybean lecithin, and the adverse impacts of seminal plasma enzymes on sperm quality during the processes of chilling and cryopreservation. Significant progress has been made in the fields of molecular biology namely lipidomics, proteomics, metabolomics, DNA methylation providing valuable knowledge regarding the unique reactions of sperm to cryopreservation. The utilization of the “*omics*” technologies has shown intricate molecular transformation that occur in sperm during freezing and thawing. Moreover, detection of molecular biomarkers that indicate the quality of sperm and their ability to withstand freezing provides opportunities to choose the best sperm samples for cryopreservation. This, in turn, enhances the results of artificial insemination and genetic conservation endeavors. This review emphasizes the necessity for adopting a comprehensive approach that combines molecular and cellular knowledge with practical methods in the field of sperm cryopreservation to ensure production of goats as major food animals in the global scale.

## 1 Introduction

Goats, among the first domesticated animals, have coexisted with humans for thousands of years (1). They are globally distributed due to their adaptability to various climates (2). Primarily valued for meat, milk, fiber, and skin, goats are especially crucial where land is scarce (3). They are present on all continents except Antarctica, demonstrating their adaptability (4). Their importance to small-scale farmers, particularly those in rural areas with limited resources, is significant due to their small size and adaptability. Global goat population has surpassed one billion, doubling in the past 30 years due to increased use by smallholder farmers (5, 6). Advances in genetics have improved goat farming efficiency, focusing on meat and milk production (7). Sustainable farming must integrate productivity, animal health, and food security through public-private partnerships and research (8). Therefore, enhancing these practices can significantly benefit sustainable farming. As such, goats are vital globally, and addressing challenges is crucial for improving nutrition and economic stability through food animals (9).

Buck fertility is the ability of a male goat to produce viable sperm capable of successful fertilization, which is essential for goat reproduction and affects herd productivity as well as genetic progress. Male fertility in artificial insemination programs is linked to sperm fertilizing capability and genetics. Alpine bucks are recognized for their superior traits such as better semen volume, concentration, and motility, making them ideal for breeding programs (10). Even bucks that pass fertility tests may exhibit subfertility (11), which necessitates detailed sperm structure and function analysis. Bucks also induce estrus in does, enhancing overall herd productivity (12). Artificial insemination with frozen semen has improved performance in Alpine goats, showcasing the importance of superior genetics (13). Selecting high-quality bucks is crucial for improving fertility (14). Artificial insemination with frozen-thawed semen has been successful in maintaining viable sperm and achieving good conception rates (15). It has also been effective in enhancing breeding outcomes in synchronized goats (16). Nutritional and environmental factors, such as seasonal variations and heat stress, significantly impact buck fertility. Proper nutrition with balanced protein and energy supports testicular size and sperm production (17) while heat stress can lead to DNA damage and altered gene expression, and reducing fertility (18, 19). Managing these factors and including in the buck soundness exam are critically important for maintaining fertility and productivity.

Cryobiology examines the limits of life under freezing conditions and how organisms can be preserved for revival. The critical method of cryopreservation involves preserving biological materials, including organelles, for long periods of time at low temperatures. This technique is essential in fields such as artificial insemination, organ transplantation, and long-term cell storage (20–22). Cryoprotectants prevent formation and damaging effects of ice crystals on molecular and cellular anatomy of sperm during freezing and thawing (23), with natural examples seen in species such as frogs that produce cryoprotectants to survive winter (24, 25). Alaska wood frogs, for instance, synthesize higher amounts of cryoprotectants to survive freeze-thaw cycles (26). Cryopreservation is also used as a conservation tool for endangered species such as the Louisiana pine snake, where sperm viability post-thaw has been studied (27). Cryopreservation is also utilized for microbial organisms in teaching, research, and industry (28). Sperm cryopreservation allows superior donor genetics to be used in artificial insemination programs (29), with slow freezing and vitrification as common methods. New cryoprotectants such as trehalose enhance cell preservation (23, 30). Age affects sperm quality during cryopreservation, with older bulls showing reduced motility and oxidative damage (31). Cryoprotectants combined with antioxidants preserve sperm integrity (32–34), and advanced computational tools help improve post-thaw sperm quality (35, 36). Studying the presence of gene products and protein expression in cryopreserved sperm provides insights into fertility pathways and the potential effects of cryopreservation on gene expression after fertilization (37, 38). Supplementing extenders with trehalose improves buck sperm freeze-thawing tolerance (39, 40), and shows promise for improving sperm cryosurvival in bulls, bucks, and rams (41, 42).

## 2 Extenders for cryopreservation of mammalian sperm

Semen extenders are vital for sperm preservation, supporting fertilization by maintaining sperm metabolism, regulating pH, preventing bacterial contamination, and reducing cryogenic damage (43, 44). They regulate pH (45), serve as an energy source (46), provide antioxidant support (47), contain antibiotics to prevent contamination (48), and help mitigate freezing shock (49). Extenders are used for both short-term chilling and long-term cryopreservation (50), with common ingredients such as egg yolk, skimmed milk (51), and plant-based alternatives like soybean lecithin (52). Egg yolk extenders form complexes with Bovine Seminal Plasma proteins (BSP proteins) to preserve sperm motility (53) while skimmed milk regulates pH and chelates heavy metals (54). Honey, due to its hyperosmotic properties, enhances sperm motility and reduces abnormalities (55, 56). Fish oil, incorporated into extenders, improves semen performance during freeze-thawing and artificial insemination (43). Soybean lecithin is a sanitary alternative, protecting against freezing shock (57).

Cryopreservation stimulates Reactive Oxygen Species (ROS) generation, such as superoxide anions ($O_{2}^{-}$), hydrogen peroxide (H_2_O_2_), and hydroxyl radicals (OH•) that trigger lipid peroxidation (58, 59) and cause irreversible damage to sperm membranes, mitochondria, and DNA. Superoxide dismutase (SOD), a defense enzyme, catalyzes the conversion of $O_{2}^{-}$ to H_2_O_2_ and O_2_ to avert mitochondrial dysfunction and premature capacitation (60). Yet, in the event of no neutralization, H_2_O_2_ is implicated in the Fenton reaction, forming highly reactive OH• in the presence of Fe^2+^ that initiate lipid peroxidation by targeting polyunsaturated fatty acids (PUFAs) such as docosahexaenoic acid (DHA) in sperm membranes, resulting in loss of membrane integrity, impaired motility, and DNA fragmentation (61, 62).

At the molecular level, antioxidants such as Epididimal Glutathione Peroxidase 5 (GPx5) play a critical role in detoxifying reactive intermediates and protecting DNA, lipids, and proteins from oxidative damage (63). At the cellular level, enzymatic antioxidants (e.g., SOD, catalase) and non-enzymatic antioxidants (e.g., vitamins C and E) help preserve mitochondrial function, essential for ATP production and motility (64, 65). To counter the accumulation of H_2_O_2_ two of the main intracellular protective enzymes, catalase (CAT) and glutathione peroxidase (GPx), are able to degrade H_2_O_2_ into water and O_2_ (66) or utilize glutathione (GSH) to reduce both H_2_O_2_ and lipid hydroperoxides (LOOH), thus successfully targeting the peroxidation chain reaction. SOD is the first line of defense against ROS, specifically $O_{2}^{-}$ radicals. It catalyzes the dismutation of $O_{2}^{-}$ into H_2_O_2_ and molecular oxygen, thereby reducing the potential for cellular damage caused by these radicals (67). Adding SOD to semen extenders enhances sperm quality across species in a concentration-dependent manner. In bulls, 100–200 IU/mL SOD improves post-thaw motility and viability (68). In rams, 1–2 mM/mL SOD enhances sperm quality in both frozen and chilled semen (69). In boars, 150–300 IU/mL SOD, alone or with catalase, increase motility, viability, and embryo production while reducing ROS (70). CAT complements the action of SOD by converting H_2_O_2_, a byproduct of $O_{2}^{-}$ dismutation, into water and oxygen. This enzymatic activity is vital in preventing oxidative damage to sperm membranes and DNA. Research indicates that CAT, along with SOD, is crucial for maintaining sperm motility and viability under oxidative stress conditions (71). Adding CAT to semen extenders improves sperm quality in a concentration-dependent manner across species. In bulls, 100–200 IU/mL CAT in tris-egg yolk extenders enhances sperm viability post-thaw, while no significant effect is observed in citrate-egg yolk extenders (72). In boars, 400 U/mL CAT has been reported to improve total sperm motility, while 200–400 U/mL reduces H_2_O_2_ levels (73). In rams, 50 mM trehalose alone or in combination with 400 μg CAT has been reported to improve post-thaw sperm motility (74). In humans, 200–400 IU/mL CAT has been reported to reduce ROS levels and protect sperm viability, motility, mitochondrial membrane potential, and DNA integrity during cryopreservation (75). Following this, CAT and GPx further detoxify H_2_O_2_, converting it into water and oxygen. CAT is particularly effective at high concentrations of H_2_O_2_, while GPx utilizes glutathione to reduce H_2_O_2_ and lipid hydroperoxides, thus preventing lipid peroxidation (76). Adding GPx to semen extenders enhances sperm quality in a species- and concentration-dependent manner. In stallions, 1–5 IU/mL GPx improves acrosome integrity post-thaw but has no significant effect on motility parameters (77). During chilled storage, supplementation with 15 IU/mL GPx, along with SOD and CAT, preserves motility and viability while reducing caspase-3 activation and DNA fragmentation (78). In bulls, 1.0 mM GPx in a nano lecithin-based extender improves plasma membrane integrity, reduces lipid peroxidation, and enhances blastocyst formation *in vitro* (47). The interplay between these enzymes is crucial for maintaining cellular redox balance, especially in spermatozoa, which are particularly susceptible to oxidative stress due to their high polyunsaturated fatty acid content in membranes (79). Collectively, these enzymes work synergistically to preserve the sperm's redox balance while preventing oxidative damage and maintaining acrosomal integrity, mitochondrial activity, and fertilization ability (80, 81). Since cryopreservation significantly depletes endogenous antioxidant activity, potent antioxidant supplementation in extenders could provide a breakthrough in improving buck sperm cryosurvival and post-thaw fertilization potential (82, 83).

Vitamins C and E have also been shown to donate some electrons and provide powerful antioxidant effects by neutralizing free radicals. Selenium and zinc are equally essential as vitamins in maintaining the antioxidant defense system and facilitating the action of enzymatic antioxidants. Such minerals act as coenzymes in biochemical cascade reactions of multiple metabolic enzymatic reactions and play a significant role in the overall antioxidant defense system. Beta-carotene and lycopene are also parts of this family, providing strong antioxidant activity in protecting lipids from peroxidation. In addition, flavonoids, which are largely available in fruits and vegetables, possess antioxidant properties and lead to reduced oxidative stress (84). Adding antioxidants to semen extenders enhances post-thaw sperm quality in a species- and concentration-dependent manner. In stallions, α-tocopherol (0.5–2 mM) reduces lipid peroxidation, while ascorbic acid (0.9–1.8 g/L) improves membrane integrity and stability (85). In Bhadawari bulls, vitamin E (5 mM) and vitamin C (5 mM) individually improve post-thaw sperm parameters, with their combination (5 mM + 5 mM) providing the highest protection against oxidative stress and cold shock (86). In rams, 0.3 mM α-Tocopherol significantly improves post-thaw motility, viability, normal sperm percentage, and functional integrity, while low-dose vitamin C (0.1 mM) also enhances sperm quality but leads to more secondary abnormalities (87).

These mechanisms also protect the lipid-rich sperm membrane, composed of polyunsaturated fatty acids, from oxidative damage caused by lipid peroxidation, thereby ensuring the integrity and functionality of sperm (88). Commercial extenders such as Triladyl^®^, Ovipro^®^, AndroMed^®^, and Steridyl^®^ are widely used, with plant-based extenders minimizing contamination risk (89–91). Nanoparticles improve sperm cryopreservation by mitigating oxidative stress through their antioxidant properties, scavenging ROS, and stabilizing cell membranes (92, 93). Metal-based nanoparticles, such as zinc oxide (ZnO) and selenium oxide (SeO), enhance sperm motility, plasma membrane integrity, and DNA stability while reducing oxidative damage markers such as malondialdehyde (94, 95).

Lipid-based nanoparticles further improve post-thaw sperm quality by providing a protective barrier and stabilizing cellular membranes (96). Additionally, nanoparticles facilitate sperm purification and advanced techniques such as chromatin protection and selective sperm population enrichment, supporting innovative reproductive applications (97, 98). Liposomes, as phospholipid bilayer vesicles, enhance sperm cryopreservation by encapsulating antioxidants and cryoprotectants, thereby reducing oxidative stress, stabilizing plasma membranes, and improving post-thaw sperm viability (92, 99). They also offer alternatives to animal-derived extenders such as egg yolk, addressing biosecurity and ethical concerns, while liposome-based formulations with trehalose further improve the rheological properties of cryopreservation media (100, 101). These multifaceted roles position liposomes as a key innovation in reproductive biotechnology, enhancing sperm functionality across species (102, 103).

Success of sperm cryopreservation is significantly influenced by genetic variations, as molecular markers have been linked to semen freezability. For instance, in boars, specific genetic markers have been associated with variations in post-thaw semen quality, underscoring the role of genetics in cryopreservation outcomes (104). The composition of sperm membranes is also critical, as changes during cryopreservation, such as the loss of membrane sterols, can compromise sperm structural integrity and fertilizing potential (105–107). The use of cryoprotectants during freezing helps minimize cryoinjury and cold damage, improving motility after thawing (108). Molecular markers such as heat shock proteins 70 (HSP70) and peroxiredoxin 6 (PRDX6) have been proposed as indicators of sperm freezability, with higher levels correlating with improved semen quality and fertility (109, 110).

Metabolomic approaches revealed seminal plasma metabolites associated with sperm cryo-tolerance, offering potential for optimizing cryopreservation techniques (111). Sperm morphology, particularly flagellum size, has been linked to freezability, with larger flagella being associated with reduced cryosurvival (112). Cryopreservation impacts sperm cellular anatomy and physiology, including changes in sperm characteristics and mRNA downregulation, which are vital for fertilization and early embryo development (95, 113). Additionally, the utilization of supervised learning methods has been suggested for characterizing sperm population structures related to freezability, providing insights into the factors influencing sperm cryo-survival (114). Supervised learning, a branch of artificial intelligence, relies on training algorithms with labeled data to detect patterns and predict outcomes from new datasets. Techniques such as deep learning models, such as convolutional neural networks (CNNs), have been applied to sperm analysis, providing more accurate and consistent evaluations of morphology and motility compared to traditional methods (115–117). Furthermore, Artificial intelligence-powered systems enable rapid analysis of large datasets, facilitating predictions of sperm fertilization potential and identifying factors affecting sperm cryo-survivability (114, 118, 119). In sperm cryopreservation, supervised learning algorithms predict post-thaw motility and viability by leveraging large datasets, which helps refine freezing protocols and improve success rates in artificial insemination (116, 120).

Cryopreservation significantly impacts sperm physiology, including motility, viability, capacitation, acrosome reaction, and fertilization potential in mammals. Motility is critical for sperm function in the female reproductive tract, and it can decrease from 90 to 95% pre-cryopreservation to around 75% post-thaw in bulls (121). This process also compromises acrosome integrity and reduces fertilization success due to impaired acrosome reactions humans and alpacas (122, 123). Cryopreservation induces oxidative stress, DNA damage, and changes in gene expression, which reduce embryo quality and development (124, 125). These changes can influence gene expression that may compromise the quality and developmental potential of embryos fertilized with cryopreserved sperm. The negative impact can potentially be due to modifications in sperm proteins induced by the cryopreservation process as well (126). Furthermore, the use of cryopreserved sperm can reduce fertilization rates and blastocyst formation in *in vitro* fertilization (IVF) and intracytoplasmic sperm injection (ICSI) procedures (127).

Premature capacitation and increased DNA fragmentation are linked to oxidative damage and mitochondrial dysfunction (128, 129). Structural integrity of the sperm plasma membrane is significantly compromised during the freeze-thaw cycles, primarily due to osmotic stress, which causes mechanical strain and destabilization of the membrane. Although cryoprotectants such as glycerol provide partial protection against these damages by reducing ice crystal formation and osmotic imbalances, they are not entirely effective in preserving the full functionality and structural integrity of the membranes (130–133). Additionally, cryopreservation alters sperm's epigenetic landscape, including DNA methylation, potentially affecting gene regulation in embryos (134). These disruptions highlight the need for improved cryopreservation protocols to maintain sperm function and embryo development in Assisted Reproductive Technologies (ARTs).

## 3 Extenders for cryopreservation of buck sperm

Spermatogenesis is a complex and essential process for sexual reproduction, where diploid spermatogonia undergo proliferative divisions and differentiate into mature haploid spermatozoa through meiotic phases and sperm cell maturation (135). This transformation occurs within the seminiferous epithelium in regulated cycles in the buck testes. Initially, round spermatids are formed and then elongated along Sertoli cells to produce elongated spermatids. This process results from coordinated interactions among Sertoli cells, type A and B spermatogonia, and primary spermatocytes, which eventually develop into haploid spermatids. Spermatogenesis in bucks typically spans 47.7 days, culminating in the release of elongated spermatids into the seminiferous tubules, where histones are replaced by protamines (136). Buck sperm exhibit unique molecular characteristics, including distinct lipid compositions and proteomic profiles, which influence cryopreservation outcomes, motility, and viability (111, 137, 138). The sperm morphology, particularly the dimensions of the head and midpiece, differs from those of other mammals (Table 1) (139, 140). Regulation of gene expression during spermatogenesis is controlled by transcriptional, post-transcriptional, and epigenetic mechanisms, which are essential for sperm maturation and function (141).

**Table 1:** Organismal diversity and similarity.

**Organisms**	**Buck (*Capra hircus*)**	**Bull (*Bos taurus*)**	**Ram (*Ovis aries*)**	**Pig (*Sus scrofa*)**	**Horse (*Equus caballus*)**	**Human (*Homo sapiens*)**	**References**
Chromosome numbers	60 (29 pairs autonomous and 2 sex chromosomes)	60 (29 pairs autonomous and 2 sex chromosomes)	54 (26 pairs autonomous and 2 sex chromosomes)	38 (18 pairs autonomous and 2 sex chromosomes)	64 (31 pairs autonomous and 2 sex chromosomes)	46 (22 pairs autonomous and 2 sex chromosomes)	(205–210)
**Genes**							
- Genome size (gb)	2.9	2.8	2.7	2.5	2.5	3.1	(205–210)
- Genes and pseudogenes (count)	28,908	37,073	35,057	30,334	33,146	59,652	
- All transcripts (count)	48,672	80,267	92,176	78,200	77,102	185,363	
- mRNAs (count)	42,674	64,928	76,688	63,562	60,887	136,181	
- CDSs (count)	42,836	65,084	76,701	63,562	60,900	136,194	
- Protein coding (count)	20,755	21,677	21,300	20,790	21,129	20,080	
Puberty	4–6 months	8–12 months	4–6 months	5–6 months	12–24 months	9–14 years	(211–215)
Spermatogenesis (days)	47.7	61	45-49	41	57	74	(216–218)
Seminiferous epithelium cycle (days)	10.6	13.5	10.4	12	12.2	16	(217, 219, 220)
Ejaculate volume (mL)	0.5–1.5 (average)	5–8 (average)	0.5–2	85–200	50–130	2–5	(212, 221–225)
Sperm/ejaculate	2–4 × 10^9^/mL (average)	1–1.5 × 10^9^/mL (average)	2–4 × 10^9^/mL (average)	200–400 × 10^6^/mL (average)	200–400 × 10^6^/mL (average)	15–200 × 10^6^/mL (average)	(226–231)
**Head**							
- Area (μm^2^)	29.8–29.9	38.05–38.15	34.90–34.95	34.8–34.9	11.43	9.25–9.27	(139, 232, 233)
- Perimeter (μm)	22.1–22.2	25.7–25.8	23.65–23.75	23.9–23.95	13.76	11.75–11.85	
- Width (μm)	4.25–4.35	4.65–4.75	4.8–4.9	4.55–4.65	2.79	2.55–2.75	
- Length (μm)	8.15–8.25	9.45–9.55	8.55–8.65	8.8–8.9	5.35	4.2–4.3	
Middle piece (μm)	12.6–12.8	13.25–13.95	14.03	16.20–16.25	10.08–10.22	1.62–2.63	(140, 234–237)
Tail (μm)	50.3–50.7	61.1–61.7	No information	62.4–68.4	48.52–49.08	36.18–49.75	(140, 234, 236, 238)
**Nucleus**							
- Area (μm^2^)	25.52	33.80	31.3–31.7	27.1–27.4	No Information	~12–15^a^	(139, 239, 240)
- Perimeter (μm)	20.75	24.35	22.5–22.7	21.75–21.95		~14^a^	
- Width (μm)	4.09	4.71	4.76–4.8	4.08–4.12		3.26–3.3	
- Length (μm)	7.80	9.12	8.3–8.4	8.22 ± 0.05		4.47–5.03	
Acrosome area (μm^2^)	21.2–25.2	23.20	26.10–26.18	29.9–34.50	12.13–14.04	Acrosome coverage (%) 46.29 ± 8.63	(140, 233, 241–243)

a Estimated area and perimeter dimensions according to width and length (240).

Cryopreservation-induced sperm damage extends beyond oxidative stress and membrane destabilization, significantly compromising DNA integrity through multiple mechanisms. The primary causes of DNA fragmentation in buck sperm include species-specific genetic characteristics, osmotic stress, and apoptotic-like pathways, differing in severity from human and cattle sperm (142, 143). Buck sperm is particularly vulnerable due to its high PUFA content, which makes it more susceptible to lipid peroxidation-derived aldehydes such as malondialdehyde (MDA), further exacerbating DNA fragmentation (144, 145). Additionally, buck sperm chromatin is less compact and retains more histones than human and bovine sperm, increasing its susceptibility to shearing forces during freezing-thawing (146, 147). Osmotic stress during cryopreservation induces torsional strain on DNA, leading to increased single- and double-strand breaks (148, 149). Moreover, mitochondrial apoptotic pathways may be triggered, where excessive ROS accumulation activates caspases and endonucleases, further degrading DNA—an effect more pronounced in bucks due to their higher metabolic activity and mitochondrial ROS production (150, 151). These vulnerabilities necessitate cryoprotectant strategies focused on DNA preservation, incorporating antioxidants such as GSH, vitamin E, polyphenols, seleno-organic molecules, and flavanoids which have been shown to mitigate cryo-induced genetic damage (152–156). Given these unique susceptibilities, buck sperm cryopreservation requires distinct DNA optimization strategies compared to human and bovine sperm.

Buck sperm exhibits distinct physiological characteristics compared to bull sperm, particularly in proteomic composition, motility, and reproductive performance (8). Zhu et al. (147) identified 238 differentially abundant proteins in buck sperm, involved in energy production and oxidative stress mitigation. Cryopreservation significantly reduces buck sperm motility and viability, primarily due to diminished membrane integrity and mitochondrial dysfunction, which impair energy production and flagellar movement (157, 158). Capacitation and acrosome reactions in buck sperm differ from that of bull sperm in that they occur more rapidly, are more sensitive to environmental conditions such as pH and ionic changes, show greater vulnerability to oxidative stress, and experience increased plasma membrane damage during cryopreservation, affecting fertilization success. Because of these differences, extenders as well as the processes of freezing and thawing might require refined compositions and methods to optimize outcomes (147). Nutritional factors, including dietary supplementation, further influence buck sperm quality, improving motility and membrane integrity (157). As such, further research is needed to understand better the unique physiological and molecular adaptations of buck sperm, particularly in response to oxidative stress, cryopreservation, and environmental factors affecting fertilization success.

Researchers employing proteomic and lipidomic analyses have identified key molecules affecting goat sperm viability after cryopreservation (138, 159). Heat shock proteins such as HSP70 and HSP90 prevent protein denaturation during freezing, while antioxidant enzymes such as SOD and catalase protect against oxidative stress (160, 161). Proteins such as proline dehydrogenase (PRODH) enhance membrane stability by scavenging ROS and supporting cellular structures, while ubiquinone (coenzyme Q10) acts as an antioxidant to reduce oxidative stress and preserve mitochondrial function, significantly improving motility and membrane integrity (29, 162). Differentially expressed proteins (DEPs) linked to energy metabolism and oxidative stress response further contribute to sperm cryotolerance (138). The fluidity of goat sperm plasma membranes is greatly decreased after epididymal maturation, which is characterized by higher lipid phase fluidity in caput (immature) than in cauda (mature) sperm membranes, as measured with pyrene and 1,6-Diphenyl-1,3,5-Hexatriene (DPH) lipid descriptors. Cholesterol also enhances membrane stability and reduces cryoinjury, supporting sperm viability post-thaw (163). Future research should quantify specific alterations in lipid and protein profiles to refine cryopreservation protocols and optimize reproductive outcomes in goats.

**Figure 1:**
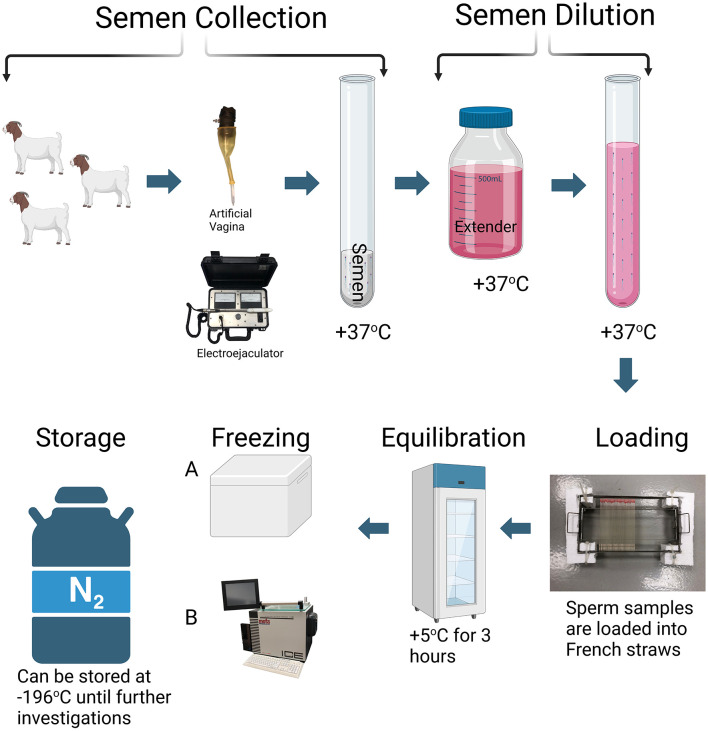
Sperm cryopreservation protocol. The cryopreservation process of semen typically begins with the collection of semen using an artificial vagina. However, electroejaculation may be employed in cases where elite male animals are unable to mount due to conditions such as lameness, shyness, or limb abnormalities. The collected ejaculate is then evaluated for key quality parameters such as motility and morphology. Following this assessment, semen is diluted with appropriate extenders. The diluted semen is loaded into 0.25 or 0.5 mL straws and cooled to 5°C, followed by an equilibration at this temperature for ~3 h. During the freezing stage, temperature reduction occurs in distinct phases to minimize cellular damage and osmotic stress. Initially, the straws are cooled to −5 to −7°C to induce extracellular ice nucleation, preventing intracellular ice formation. This is followed by a controlled cooling phase, during which the temperature is gradually reduced at a rate of ~1°C per min until reaching −35°C. This step allows the cells to adapt to the temperature decrease, minimizing intracellular crystallization and membrane damage. The final phase involves rapid cooling to −196°C, which transitions the sperm cells into a vitrified state, halting all metabolic activities and preserving cellular integrity (204). Two main methods are employed during the freezing stage of cryopreservation. The first, conventional method involves placing the straws in a styrofoam box, 4–8 cm above the liquid nitrogen surface (with a nitrogen level of 5 cm) to be frozen in the nitrogen vapor for 15 min (Method A). The second method utilizes automatic freezing systems. These systems offer a wide range of temperatures, from +40°C to −180°C, and operate with precise cooling rates between 0.01°C and 60°C/min (Method B). Both methods aim to ensure the gradual adaptation of sperm cells to temperature changes, thus preserving their viability and motility. Following cryopreservation, the straws are transferred into liquid nitrogen (−196°C) for long-term storage.

Cryopreservation of buck sperm is absolutely important for both fundamental and applied animal reproduction science (Figure 1), allowing long-term storage and transport of the genetic material. Semen collection methods significantly impact sperm cryoresistance, with different techniques influencing sperm survival during cryopreservation (164). Extenders such as Tris-egg yolk-glucose and non-fat dried skimmed milk are commonly used to protect sperm during freezing and thawing (165). Tris-egg yolk-glucose provides essential nutrients and energy, while non-fat dried skimmed milk supplies proteins and lipids necessary for maintaining sperm viability. Glycerol prevents ice crystal formation, which is the key to preserving cellular integrity during freezing (166). Cholesterol-loaded cyclodextrins improve membrane integrity, increasing both viability and motility in buck and bull sperm (167). Fruit juices, such as pineapple (Ananas comosus), orange (Citrus sinensis), and cucumber (Cucumis sativus), contain antioxidants like carotenoids, vitamins (C and E), phenolic compounds, and flavonoids, which have been shown to enhance motility and reduce sperm abnormalities by mitigating oxidative stress (168). The success of buck sperm cryopreservation largely depends not only on the extender composition, as each component plays a crucial role in protecting sperm cells, but also on the characteristics of the ejaculate itself, including the presence of seminal plasma and seasonal variations in sperm freezability throughout the year (169, 170). For instance, cholesterol-loaded cyclodextrins improve membrane integrity, leading to better post-thaw viability (167). Metabolomics studies reveal that trehalose supplementation (60–100 mM) enhances post-thaw sperm motility, viability, mitochondrial activity, and DNA integrity when combined with low concentrations of cryoprotectants (36, 171). Trehalose exerts its cryoprotective effects by stabilizing sperm plasma membrane phospholipids, preventing cellular dehydration, and reducing lipid peroxidation, thereby minimizing oxidative stress and preserving sperm function after cryopreservation (172).

**Figure 2:**
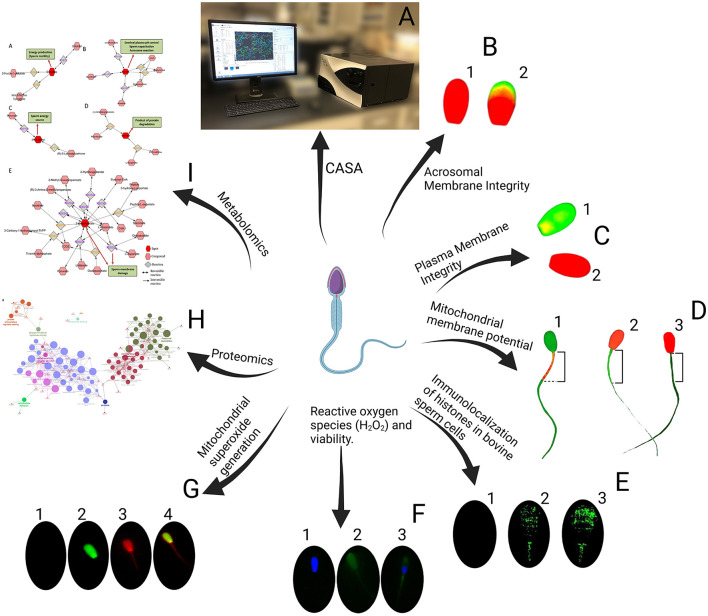
Sperm evaluation. **(A)** CASA (Computer-Assisted Sperm Analysis). **(B)** Acrosomal Membrane Integrity, B1: Intact acrosomal membrane, B2: Damaged acrosomal membrane. **(C)** Plasma Membrane Integrity, C1: Intact plasma membrane, C2: Damaged plasma membrane. **(D)** Mitochondrial membrane potential, D1: High mitochondrial membrane potential, D2: Low mitochondrial membrane potential, D3: No mitochondrial membrane potential. **(E)** Immunolocalization of Histones in Bovine Sperm Cells, E1: Negative control sperm cell incubated with only the secondary antibody, E2: Sperm cell with a medium level of histone fluorescence, E3: Sperm cell with a high level of histone fluorescence. **(F)** Reactive Oxygen Species (H_2_O_2_) and Viability, F1: Viable sperm without ROS, F2: Dead sperm with ROS, F3: Viable sperm with ROS. **(G)** Mitochondrial $O_{2}^{-}$ generation, G1: Live cells without stain, G2: Dead cells with green stain, G3: positive mitochondrial $O_{2}^{-}$ generation, G4: Dead and positive mitochondrial $O_{2}^{-}$ generation. **(H)** Proteomics. **(I)** Metabolomics.

Cryopreservation of buck sperm presents challenges in maintaining motility, plasma and acrosomal membrane integrity, mitochondrial membrane potential, and reducing ROS generation. Additionally, it leads to proteomic and metabolomic alterations due to structural damage, ultimately lowering sperm viability (171) (Figure 2). The choice of extender is a critical factor, as different extenders affect sperm quality in various ways (Table 2) (173). Sperm quality is also influenced by age, and supplementation with antioxidants has shown potential in improving these parameters (174). Re-adding seminal plasma post-thaw has had limited success, indicating the need for novel approaches (175). Innovative additives such as Mito-TEMPO have shown promise in improving sperm cryopreservation (176). Cholesterol supplementation has also been effective in enhancing cryosurvival (177). Factors such as semen collection methods, extender choice, and centrifugation all influence post-thaw sperm quality, emphasizing the need for standardized protocols (165). Antioxidant supplementation, such as cysteine, improves motility and viability, highlighting its potential in enhancing cryosurvival (178). Studies on tris and egg yolk concentrations further emphasize the importance of extender composition for post-thaw viability (179). Soy-based extenders have shown promise in improving semen freezability (180).

**Table 2:** Goat semen preservation and efficiency.

**Study**	**Breed**	**Diluent**	**Supplement**	**Primary outcome**
Kumar et al. (82)	Black Bengal buck	Tris-Egg Yolk-Citrate extender	Control T1—Vitamin E, 3 mmol/mL (Vit-E) T2—Quercetin 10 μmol/mL T3—Quercetin 20 μmol/mL T4—Quercetin 30 μmol/mL	Quercetin at 20 μmol/mL improved post-thaw sperm plasma membrane integrity, viability, acrosomal integrity, mitochondrial membrane activity, and sperm kinematics. It also significantly reduced ROS and RNS levels, increased antioxidant enzyme activity (CAT, SOD, GPx, FRAP), and lowered lipid peroxidation (MDA levels). Higher total and progressive motility observed at 20 μmol/mL quercetin compared to control.
Batool et al. (83)	Crossbred Kamori goats	Tris-egg yolk extender	Control Quercetin 1 μmol/mL Quercetin 5 μmol/mL Quercetin 10 μmol/mL Quercetin 15 μmol/mL	Quercetin at 5 μM improved post-thaw sperm total and progressive motility, plasma membrane and acrosome integrity, and viability. It also significantly increased antioxidant enzyme activity (SOD, CAT, POD, APX, TAC) and reduced oxidant levels (TOS, MDA). Pregnancy rate was higher in the quercetin-treated semen group (80%) compared to control (60%).
Suwor et al. (244)	Anglo-Nubian goats	Tris, Citric acid, Glucose, Calcium chloride	Exp. 1. Glycerol (5%) combined with soybean lecithin (1 and 3%) and egg yolk (10 and 18%). Exp. 2. soybean lecithin (3%) and glycerol (5%) with 1, 3, and 5 mM for Glutathione or 6, 9, and 12 mM for Cysteine or 1, 2, and 3 mM for vitamin E	Exp. 1.—Highest motility (44.70 ± 0.57%) and viability (53.00 ± 0.37%) with 5% glycerol and 3% soybean lecithin. No significant difference in DNA methylation with glycerol (5%) combined with either soybean lecithin (3%) or egg yolk (10%) compared to fresh sperm. Exp. 2.—Addition of 5 mM glutathione significantly enhanced frozen sperm quality (motility, viability, acrosome integrity, membrane integrity, and mitochondrial activity). 5 mM glutathione or 6 mM cysteine reduced lipid peroxidation of frozen semen.
Ali et al. (245)	Kamohri buck	Tris-based Egg Yolk (TEY) extender	Selenium at concentrations of 0 mM (Group A, control), 2 mM (Group B), 4 mM (Group C), and 6 mM (Group D).	*In-vitro*: Group B showed significantly higher motility, morphology, membrane integrity, and live-dead ratio both chilled and post-thaw. *In-vivo*: Group B (2 mM selenium supplementation) had a significantly higher conception rate (50%) compared to Group A (control, 30%).
Bucak et al. (246)	Angora goat	Tris-based extender	Control, Lipid mixtures (Liposomes) (321.99 μg), Lipid mixtures (Liposomes) (841.33 μg), Melatonin (0.25 mM), Melatonin (1 mM), Lipid mixtures (Liposomes) (321.99 μg) + Melatonin (1 mM), Lipid mixtures (Liposomes) (841.33 μg) + Melatonin (0.25 mM)	The addition of Lps 321.99 μg/mL (65 %), gave the best motility, plasma membrane and acrosomal membrane integrity (*p* < 0.05). Lps 321.99 μg/mL + Mel 1 mM and Lps 841.33 μg/mL + Mel 0.25 mM have decreased DNA damage and abnormal DNA.
Salama et al. (247)	Boer goat bucks	Tris-based extender	0%, 5%, 10%, 15% PRP	Increased motility, viability, antioxidant activity, pregnancy and kidding rates with 10% PRP
Khalique et al. (248)	Beetal buck	TRIS-citrate-yolk	0, 25, 50, 75, 100 μg/mL CeO2NPs	Enhanced motility, viability, membrane integrity, fertility outcomes with 25 and 50 μg/mL
Saratsi et al. (249)	Skopelos bucks	OviXcell^®^	0, 2.15, 10, 30 mM Fumaric Acid	Improved viability, membrane and acrosome integrity, mitochondrial function with 2.15 mM
Galián et al. (250)	Murciano-Granadina goat	Different extenders (IMIDA, skim milk-based, etc.)	Skimmed-milk-based, SDS in egg-yolk-based (IMIDA), etc.	Highest sperm quality with new IMIDA extender
Shah et al. (251)	Beetal buck (Capra hircus)	Triladyl^®^	T1: 10% v/v, T2: 15% v/v, T3: 20% v/v egg yolk in Triladyl^®^	Optimum fertility rate (73.53%) with 10% v/v egg yolk in Triladyl^®^
Ghanem et al. (252)	Buck	83 mM citric acid, 250 mM tris-hydroxymethyl- aminomethane, 50 mM glucose, 0.1 M sucrose, 3 M dimethyl sulfoxide	Melatonin (M), L-carnitine (LC), cysteine (Cys), LC + M, M + Cys, LC + Cys, LC + Cys + M	Improved post-thaw physiochemical properties with Cys alone or in combination with LC
Liang et al. (253)	Goat	Andromed^®^, Optidyl^®^, Sigma l-phosphatidylcholine, Skim milk	Egg yolk, skim milk, soybean lecithin	Improved post-thaw quality and fertility with Andr^®^ and Opt^®^
Caamaño et al. (254)	Bermeya	Tris, citric acid and glucose	Exp. 1—Control (C), 10, 50, 100 μg/mL (0, 33, 164 and 329 μM/mL) taxifolin (T) Exp. 2.—(Lower taxifolin concentration was chosen according to Exp. 1.)—C, 5 μM/mL (1.5 μg/mL) T, 1 mM GSH and combine	Exp. 1.—T10 increased progressive motility (*P* < 0.001), decreased viability on the three concentrations (*P* < 0.001), at 0 and 5 h T10 decreased Cytoplasmic ROS (*P* = 0.049), decreased mitochondrial $O_{2}^{-}$ at all doses (*P* = 0.024). Exp. 2.—5 μM taxifolin or 1 mM GSH (whether used individually or in combination) enhanced both total and progressive motility. taxifolin improved kinematic parameters such as VCL, ALH, and DNC (*P* < 0.05).
Abedin et al. (255)	Assam Hill	Tris, citric acid, fructose, EY extender	T0 (C), T1 (0.1 mg/mL ZnO NPs), T2 (0.5 mg/mL ZnO NPs), T3 (0.5 μg/mL Se NPs), T4 (1 μg/mL Se NPs)	The addition of 0.1 mg/mL Zinc Oxide Nanoparticles (ZnO NPs) to the extender significantly improved the post-thaw quality of goat spermatozoa by enhancing antioxidant enzyme activities and reducing lipid peroxidation levels.
Esmaeilkhanian et al. (256)	Saanen	Tris, citric acid, fructose, EY extender	Mito-TEMPO with doses of 0, 1, 10, 100, and 1000 μM.	Mito-TEMPO with doses of 0, 1, 10, 100, and 1000 μM. Apoptotic-like Changes and ROS Concentration: Decreased in 10 and 100 μM Mito-TEMPO groups. Mitochondria Membrane Potential: Higher in 1, 10, and 100 μM Mito-TEMPO groups. DNA Fragmentation: Lowest in the 10 μM Mito-TEMPO group.
Akhondzadeh et al. (257)	Mature goat bucks	Tris-citrate-fructose-soybean lecithin extender	Antifreeze protein (AFP) at concentrations of 0 μg/mL (A0), 5 μg/mL (A5), and 10 μg/mL (A10) combined with either 7% glycerol (G7) or 5% glycerol (G5)	Total and Progressive Motility: Higher in A5G5 and A5G7 groups (*p* < 0.05). Plasma Membrane Integrity, Sperm Acrosome Integrity, DNA Integrity, Acrosome Intact Sperm, and Mitochondrial Membrane Potential: Higher in A5G5 and A10G5 groups (*p* < 0.05). Sperm Viability: Higher in A5G5 (*p* < 0.05). Lipid Peroxidation: Lower in A5G5 and A5G7 groups (*p* < 0.05). Apoptosis Occurrence: Lower in groups with 0 μg/mL AFP and higher live post-thawed spermatozoa in groups with 5 μg/mL AFP combined with either 5 or 7% glycerol (*p* < 0.05).
Karaşör et al. (258)	Ankara buck	Tris, citric acid, fructose, EY extender	ROCK inhibitor (5 and 20 μM), antifreeze protein III (1 and 4 μg/mL), boron (0.25 and 1 mM)	ROCK inhibitor and boron improved post-thaw motility (71.82 and 76.36%) compared to control (66.15%); antifreeze protein III showed minimal impact on motility (70.58%). DNA damage reduced significantly with antifreeze protein III (1.23%) and boron (1.83 and 1.18%) compared to control (3.37%). No significant effect on plasma membrane, acrosome integrity, or mitochondrial membrane potential.
Dhara et al. (259)	Pantja buck	Egg yolk-tris (EYT) extender	1%, 3%, 5%, 7%, 9% v/v BSP proteins	Improved post-thaw semen quality with 5% BSP proteins
Zhang et al. (29)	Laoshan	Tris, citric acid, fructose, EY extender	Proline at 0, 0.5, 1, 2, and 4 mM concentrations	Adding 2 mM proline to the freezing medium significantly improved the quality of post-thaw goat sperm. This improvement was marked by enhanced motility, membrane and acrosome integrity, along with increased antioxidant levels and decreased oxidative stress markers.
El-Khawagah et al. (260)	Boer and Zaraibi	Tris-based	Butylated hydroxytoluene (BHT) 0.5 mM in Tris-soya lecithin, 1.0–2.0 mM in Tris-egg yolk	BHT at 0.5 mM in Tris-soya lecithin and 1.0–2.0 mM in Tris-egg yolk improved sperm motility, plasma and acrosome membranes, and DNA integrity. Reduced lipid peroxidation at 1.0–2.0 mM.
Susilowati et al. (15)	Kacang	Egg yolk-citrate	Simmental bull seminal plasma protein 2.5 mg/mL	2.5 mg/mL Simmental bull seminal plasma protein increased post-thaw viability, motility, and intact plasma membrane. Higher conception, pregnancy, and kidding rates.
Sun et al. (261)	Chongming White goats	Tris-based	Soybean lecithin 2% SL	2% SL resulted in higher sperm viability, motility, membrane and acrosome integrity, and mitochondrial activity. Similar or better than 20% egg yolk.
Igbokwe et al. (262)	West African Dwarf (WAD) goats	Tris-based	Tiger nut milk 15% TNM	15% TNM in slow freezing enhanced motility, livability, membrane and acrosome integrity. Lower abnormality and MDA concentration.
Lv et al. (263)	Not specified	Commercial bull semen extender	Resveratrol 10, 50 μM	10 or 50 μM Resveratrol increased total and progressive motility, membrane and acrosome integrity, and mitochondrial activity. Reduced ROS production.
Sharma and Sood (16)	Chegu	Tris Citrate Egg Yolk	10% Egg Yolk, 6% Glycerol	Post-thaw sperm parameters such as motility and viability were significantly improved, leading to a conception rate of 42.5%.
Gororo et al. (264)	Small East African goat	Various extenders	Extender 1—1.38% glucose + egg yolk (18%) Extender 2—0.30% glucose + egg yolk (2.5%) Extender 3—0.20% fructose + non-egg yolk	Non-frozen semen viable up to 24 h at 4°C in low or non-egg yolk-based extenders. Higher sperm quality at lower temperature.
Pawshe et al. (265)	Malabari	Various extenders	Soybean lecithin (Bioxcell), Egg yolk (Triladyl)	Commercial egg yolk (Triladyl) based extender resulted in better cryopreservation outcomes than others.
Konyak et al. (182)	Black Bengal	Tris extender	Soybean lecithin 1% SL	1% SL maintained *in vitro* sperm characteristics similar to egg yolk, optimal for Black Bengal buck semen.
Swelum et al. (266)	Aardi	Tris, citric acid, fructose, EY extender	Chicken (C), pigeon (P), goose (G), Japanese quail (Q), duck (D), or turkey (T) egg yolks	Chicken egg yolk provided the best results for post-thaw buck semen quality, particularly in sperm motility, vitality, plasma membrane integrity, DNA integrity, and lower sperm abnormalities. It also showed the lowest malondialdehyde levels and highest reduced glutathione activities.
El-Battawy and El-Nattat (267)	Zaraibi	Tris-based	Methionine 1.5, 2.5, 5 mM	2.5 mM Methionine improved SM% and post-thawing motility.
Yousefian et al. (268)	Mahabadi	Soybean lecithin-based	CoQ10 0.5, 1, 1.5 μM	1 μM CoQ10 protected from cryoinjury, improved motility and membrane functionality.
Narwade et al. (269)	Crossbred	Tris-based with egg yolk or soybean	Trehalose 131.25 mM with/without 25% soya	Trehalose with egg yolk improved post-thaw semen quality.
Seifi-Jamadi et al. (270)	Mahabadi	Egg yolk-based with DMA (5%) or glycerol (5%)	Control, Quercetin 10, 20 μM	10 μM Quercetin with DMA improved motility and reduced lipid peroxidation.
Salmon et al. (271)	Alpine	Skim milk-based	Cholesterol-loaded cyclodextrin (CLC) (3 mg/mL, corresponding to 141 μg/mL cholesterol)	CLC treatment improved resistance to seminal plasma damage.

Cryoprotectants such as trehalose play a protective role in preserving membrane integrity during freezing and thawing (181), and using soybean lecithin instead of egg yolk provides a safer alternative for buck semen cryopreservation (182). In Markhoz goats, 50–70 mM trehalose, alone or in combination with 3–6 mM pentoxifylline (PTX), has been reported to improve post-thaw sperm motility, viability, and chromatin integrity during cryopreservation (183). In Angora bucks, 50–75 mM trehalose has been reported to improve post-thaw sperm motility, while 50 mM trehalose resulted in the lowest percentage of total abnormalities (184). Further research is needed to optimize the techniques and antioxidants to better protect sperm during and after cryopreservation (32). Removing seminal plasma through centrifugation has improved sperm quality during cryopreservation (185), and supplementation with seminal plasma later in the cryopreservation process has enhanced post-thaw sperm quality (186). Washing procedures during cryopreservation affect sperm quality, underscoring the need for standardized protocols (166). Egg yolk concentration also influences sperm quality during cryopreservation, reinforcing the importance of extender composition (187). Natural additives, such as fulvic acids (188) and black cumin seed extract (189) have shown promise in improving motility and reducing oxidative stress in buck sperm. The cryoprotective effects of fruit juices on sperm viability further suggest their potential as natural cryoprotectants (168). Morphological changes post-cryopreservation emphasize the need for careful sperm selection for successful fertilization (10). Overcoming the challenges in buck sperm cryopreservation requires optimizing extender composition, antioxidant treatments, cryoprotectants, and semen processing techniques. By improving these factors, the success of cryopreservation can be increased, providing a valuable tool for genetic preservation in breeding programs.

Sperm cryopreservation causes molecular changes effecting sperm structure, function, and fertility. These effects vary by species due to unique sperm characteristics such as size, morphology, and membrane composition (190, 191). Factors affecting cryopreservation efficacy include cooling and thawing rates, sperm origin (ejaculate or epididymal), and individual variations (192–194). Cryopreservation lowers sperm viability and motility while increasing acrosome reaction rates (195). Proteomic analyses have identified biomarkers that influence sperm cryopreservation recovery, suggesting that deeper insights into these molecular species are crucial for understanding functional preservation in frozen-thawed sperms. Cryopreservation impacts proteins involved in sperm motility, viability, acrosomal integrity, ATP content, and capacitation. The use of “*omics*” technologies, especially proteomics, aids in optimizing freezing-thawing protocols to maintain sperm function and fertility (145, 147, 171). Different extenders used during cryopreservation contain cryoprotectants, antioxidants, and other agents to maintain sperm viability and function (190). However, while some freezing protocols yield satisfactory post-thaw sperm survival, others may result in reduced sperm viability due to suboptimal cryoprotectant composition or freezing and thawing conditions (196).

Lipidomics has been significant for studying lipid profiles, providing insights into how variations in lipid composition affect sperm quality and cryopreservation outcomes across different species. Lipidomic analysis can help predict cryopreservation success in ruminant sperm (145, 197, 198). Targeted lipidomics has been applied to uncover semen cryotolerance-related lipid profiles in Mediterranean Buffalo bulls, demonstrating its potential in evaluating sperm quality (199). The link between lipidomics and sperm fertility has been explored, focusing on cryotolerance and semen quality, identifying potential biomarkers in the spermatozoa lipidome that could be used for selecting high-fertility doses before freezing (200). Lipidomics has revealed the lipid composition of sperm cells from various species, highlighting the role of fatty acids in sperm function (201). Recent lipidomic studies have identified significant qualitative and quantitative differences in sperm membranes among ejaculates that led to pregnancy vs. those that did not (202), providing additional evidence for this approach's potential for characterizing gamete function associated with fertility outcomes. Lipidomics has also been proposed as a method to assess the spermatozoa and seminal plasma of males for fertility prediction (203).

## 4 Conclusions and the outlook

There is a need to enhance sperm cryopreservation techniques to improve sperm survival during freezing and thawing processes and ensure successful fertilization across various species, thereby contributing to the progress of reproductive biotechnologies and conservation initiatives. Advances in molecular biology, lipidomics, proteomics, metabolomics, and DNA methylation are helpful in better understanding the male gamete during freezing and thawing, and in improving extender formulations and freezing techniques in buck sperm cryopreservation. Future advancements are expected to focus on minimizing the detrimental effects of seminal plasma enzymes through the improvement of extenders, as part of broader strategies to enhance sperm quality and viability post-cryopreservation. Furthermore, detection of biomarkers that indicate the quality of sperm and their ability to withstand freezing will aid in choosing the most suitable sperm samples for freezing. This will ultimately enhance the effectiveness of artificial insemination and genetic conservation initiatives in goats.
